# Corneal Repair and Regeneration: Current Concepts and Future Directions

**DOI:** 10.3389/fbioe.2019.00135

**Published:** 2019-06-11

**Authors:** Mohammadmahdi Mobaraki, Reza Abbasi, Sajjad Omidian Vandchali, Maryam Ghaffari, Fathollah Moztarzadeh, Masoud Mozafari

**Affiliations:** ^1^Biomaterials Group, Department of Biomedical Engineering, Amirkabir University of Technology, Tehran, Iran; ^2^Department of Tissue Engineering and Regenerative Medicine, Faculty of Advanced Technologies in Medicine, Iran University of Medical Sciences, Tehran, Iran

**Keywords:** cornea, tissue engineering, wound healing, regenerative medicine, biomaterials, immune privilege, angiogenesis, limbus

## Abstract

The cornea is a unique tissue and the most powerful focusing element of the eye, known as a window to the eye. Infectious or non-infectious diseases might cause severe visual impairments that need medical intervention to restore patients' vision. The most prominent characteristics of the cornea are its mechanical strength and transparency, which are indeed the most important criteria considerations when reconstructing the injured cornea. Corneal strength comes from about 200 collagen lamellae which criss-cross the cornea in different directions and comprise nearly 90% of the thickness of the cornea. Regarding corneal transparency, the specific characteristics of the cornea include its immune and angiogenic privilege besides its limbus zone. On the other hand, angiogenic privilege involves several active cascades in which anti-angiogenic factors are produced to compensate for the enhanced production of proangiogenic factors after wound healing. Limbus of the cornea forms a border between the corneal and conjunctival epithelium, and its limbal stem cells (LSCs) are essential in maintenance and repair of the adult cornea through its support of corneal epithelial tissue repair and regeneration. As a result, the main factors which threaten the corneal clarity are inflammatory reactions, neovascularization, and limbal deficiency. In fact, the influx of inflammatory cells causes scar formation and destruction of the limbus zone. Current studies about wound healing treatment focus on corneal characteristics such as the immune response, angiogenesis, and cell signaling. In this review, studied topics related to wound healing and new approaches in cornea regeneration, which are mostly related to the criteria mentioned above, will be discussed.

## Introduction

Diseases affecting the cornea can be either infectious or non-infectious, and both may cause severe visual impairments requiring intervention. Trachoma, onchocerciasis, corneal ulceration, corneal dystrophies, and xerophthalmia are some of the major causes of blindness worldwide (Sommer, 1982). However, the prevalence and epidemiology of corneal diseases varies from region to region. From the use of traditional eye medicines (which is now considered a significant risk factor for corneal ulceration) to collagen cross-linking, which has recently been approved by the US FDA to strengthen the cornea, the ultimate goal in corneal treatment is to employ minimally invasive procedures that can restore or preserve vision (Jeng et al., 2016). Stimulating the body's repair mechanisms is now considered to be the gold standard for the functional healing of damaged tissues and organs (Khadem et al., 2000, 2004; Zarrintaj et al., 2017). This approach is typified by corneal transplantation and tissue engineering.

**Figure 1:**
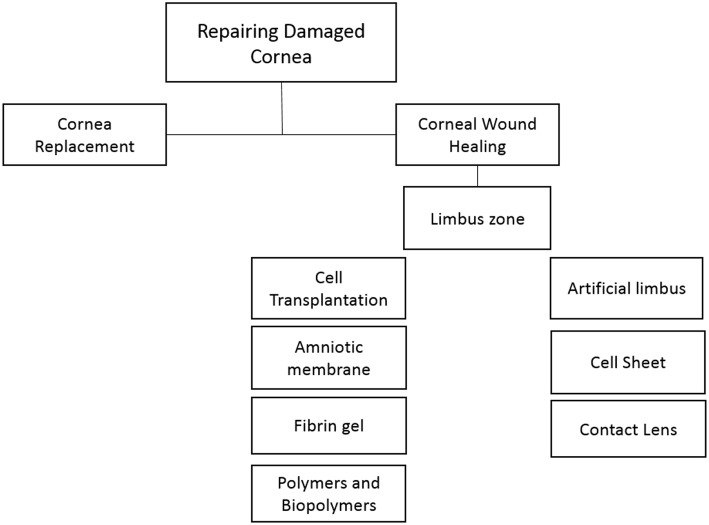
Current strategies for the repair and regeneration of damaged cornea.

The most striking advance in the medical treatment of corneal diseases over recent decades has been corneal transplantation and, nowadays, the cornea is the most commonly transplanted tissue worldwide. Corneal transplantations are divided into two main categories based upon the amount of surgically replaced tissue. In penetrating keratoplasty, the entire cornea is replaced with a donor tissue. However, in a newer procedure called lamellar keratoplasty, only the damaged layers are replaced with a donor graft, and the healthy part of the cornea is left intact. In lamellar keratoplasty, the integrity of the cornea and the surrounding tissues are preserved; as a result, better visual improvement is usually achieved. Unfortunately, there are sometimes poor outcomes because of graft rejection or late graft failure. Furthermore, according to a World Health Organization (WHO) report, 15–20% of patients who need corneal transplantation remain untreated because of the shortage of corneal donors (Whitcher et al., 2001). Over the last few decades, the shortage of donor tissues, beside fears of transmissible diseases, has accelerated studies on finding an alternative treatment to transplantation, and an artificial cornea or keratoprosthesis has been suggested as an option. Historically, Guillaume Pellier de Quengsy Jr. was the first person who proposed a thin silver-rimmed convex glass disc as an artificial cornea, as long ago as 1789 (Mannis and Mannis, 1999). At that time, the first priority was to choose transparent and non-irritating materials, but as time went by other researchers concentrated on designing an artificial cornea which was able to promote better incorporation with the host tissue. Glass and quartz were the choices for the transparent part of the prosthesis and natural polymers like gutta-percha and casein were added to the artificial cornea design. Later, gold rings and platinum rings were used to achieve better incorporation with the host cornea. Further studies on artificial corneas resulted in replacing glass and quartz with lighter materials. At the beginning of the twentieth century, attention was diverted from artificial corneas to transplantation of donor corneal after the first successful keratoplasty (Zirm, 1989). Nevertheless, studies on artificial corneas have never ceased. Today there are four types of keratoprostheses, which are in commercial use. In the following section, these keratoprostheses will be further discussed. Despite some clinical success in using artificial corneas, the host rejection is still relatively high. The presence of corneal epithelial stem cells, which are located in the basal epithelial layer of the corneal limbus (the border between the cornea and the sclera), has given some hope for better healing and integration. Therefore, many researchers have focused on employing new biomaterials to mimic the corneal architecture, which would allow better corneal self-repair. This review will discuss recent advances in repairing damaged corneas, focusing on new concepts and biomaterials (Figure 1; Muijzer et al., 2019).

## Cornea Structure and Transparency

**Figure 2:**
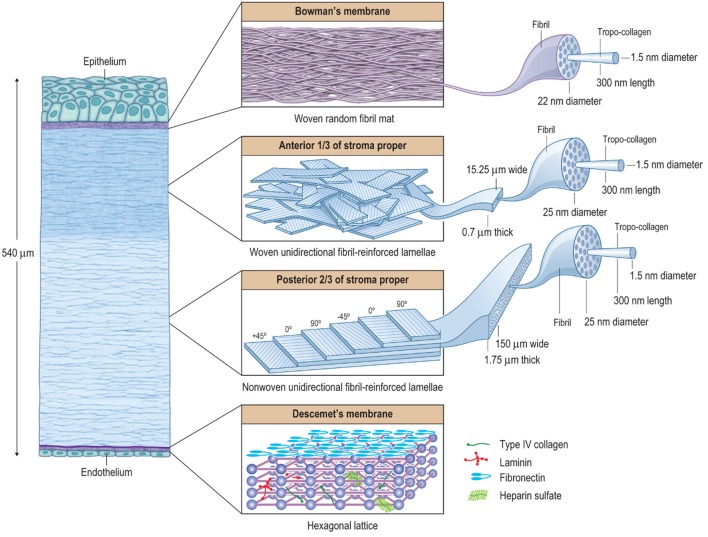
The hierarchical structure of the cornea showing that it is basically composed of three composite regions. A fourth composite region, Descemet's membrane, is included for completion. The macroscopic, microscopic, and nanoscopic features are emphasized (from left to right) to help illustrate the various interactions between the tissue components. Bowman's layer is essentially a random fibril, woven-mat composite, which maximizes multi-axial stiffness and strength. The underlying anterior third of the stroma proper is a lamellar interwoven fabric composed of unidirectionally (UD) fibril-reinforced lamellae. This architectural hierarchy is more rigid against z-axis deformations compared to non-woven UD-laminates. In the human body, the corneal structure is most similar to that of the pericardium, which serves to mechanically prevent the formation of aneurysms in the heart. The posterior two-thirds of the stroma is essentially a non-woven, UD-fibril-reinforced lamellar composite, which maximizes longitudinal x- and y-axis stiffness and strength, but has only weak transverse z-axis stiffness and strength. In the human body, its structure is most similar to that of the annulus fibrosis of the intervertebral disk, which functions efficiently as a cushioning mechanism for the spine, but is prone to chronic biomechanical failure. The UD-orientation of collagen fibrils in each lamella is vital because this arrangement prevents fibril undulation and thus maximizes the initial axial tensile strength of each fibril. Descemet's membrane forms a hexagonal lattice. Taken together these composite-like regions are responsible for the overall stiffness, strength, extensibility, and toughness of the cornea. They also help explain how the cornea behaves biomechanically after surgery, disease, or injury. Reprinted with permission (Kaufman et al., 2011).

The human cornea is a unique tissue with two critical functions. On the one hand, the cornea forms the anterior portion of the outer casing of the eye and protects the inner portion of the eye from the external environment. On the other hand, it is the single most powerful focusing element of the eye. It provides about 80% refractive power of the eye and it is roughly twice as powerful as the lens. Because of these functions, the cornea is both mechanically strong and transparent. Its strength comes from about 200 collagen lamellae, which criss-cross the cornea in different directions. This collagen-rich layer of the cornea comprises nearly 90% of the thickness of the cornea and is called the “stroma proper layer.” In fact, the human cornea is composed of five primary layers—epithelium, Bowman's layer, stroma proper, Descemet's membrane, and endothelium. This hierarchical structure can be described as a fibril-reinforced laminate biocomposite, which provides an excellent compromise between stiffness, strength, toughness, and extensibility. In Figure 2 the hierarchical structure of the cornea, with macroscopic, microscopic, and nanoscopic features, is shown (Kaufman et al., 2011). As noted earlier, the cornea is transparent; however, the hierarchical structure and the presence of interfaces between the different layers, each with its own index of refraction, seem to be in contradiction with its clarity. There have been lots of studies attempting to explain the transparency of the cornea. The first explanations in the nineteenth century emphasized the homogeneity of the cornea and the fact that the collagen fibrils had the same refractive index. After about a century, Dr. Maurice (1957) tried to explain the optical structure of the cornea considering its geometric form, dimensions and the refractive indices of its components. He suggested that the scattered waves from collagen fibrils interfered with each other in such a way that they canceled out each other in all directions, except the forward direction. Meanwhile, Miller and Benedek (1973) in 1973 showed that the gaps between the interfaces were smaller than one-half the wavelength of visible light; and as a result, the cornea is crystal clear. Recently Meek and Knupp (2015) have reviewed the current state of knowledge about the corneal architecture and its optical transparency. These authors outlined the general basis and molecular mechanisms of corneal transparency. Maurice's suggestion regarding the interaction of the incoming electromagnetic waves with the collagen fibrils was proved to be correct by new imaging technologies (Quantock et al., 2015).

## Corneal Replacements

Boston Keratoprosthesis (B-KPro), Osteo-Odonto-Keratoprosthesis (OOKP), AlphaCor, and the KeraKlear Artificial Cornea are the four types of keratoprostheses which have, so far, been commercialized. Over the last decade, implantation of artificial corneas has been dramatically increased. For instance, fewer than 50 units of B-KPro were implanted before 2002, while more than 9,000 implantations were carried out in 2014. In this section, recent advances in the design of artificial corneas will be discussed with regard to the type of materials employed.

The B-KPro is the most widely implanted artificial cornea and was developed at the Massachusetts Eye and Ear Infirmary. There are two types of B-KPro each with different indications. The B-KPro type I is commonly used in patients with a non-cicatrizing disease such as repeated allograft failure, corneal opacity with extensive neovascularization, aniridia, trauma, etc. In contrast, B-KPro type II is used in cicatrizing diseases and severe dry eye conditions, such as severe autoimmune ocular diseases. Both types of B-KPro have similar compartments but are different in some details. They have a front plate and a back plate, which act to sandwich a fresh donor cornea; a titanium locking-ring is used to secure the plates. Medical grade polymethyl methacrylate (PMMA) with the ability to block UVA/UVB is used for the front and back plates. The idea of using PMMA was conceived due to its ability to induce only minimal inflammatory responses in the eye (Griffith et al., 2016). PMMA is a transparent thermoplastic polymer, also known as acrylic glass, which can be modified to achieve desired mechanical properties, like toughness and stiffness. On the other hand, modifying the PMMA polymer with nano-dimensional TiO2, SiO2, ZnO, ZrO2, Al2O3, CNT, and graphene has been investigated for electromagnetic shielding, thermal insulation, antiglare resistance, scratch resistance, and also resistance against UV radiation (Pandey et al., 2010; Cano et al., 2013; Soumya et al., 2014). The apparent success of PMMA in keratoprostheses has been plagued by numerous complications like tissue necrosis, retroprosthetic membrane formation, vitreous opacities, etc. The main reasons for these shortcomings include poor adhesion between the PMMA and the host corneal collagen at the PMMA-host interface and fibrous membrane formation due to fibroblast attachment onto the inner surface of PMMA. Therefore, there have been ongoing research efforts to overcome these drawbacks. In the first attempts, PMMA surfaces were modified with polyethylene glycol (PEG) or extracellular matrix molecules to block cell adhesion (Kim et al., 2001; Aucoin et al., 2002). The results were promising in terms of preserving the inner surface of PMMA but, at the external PMMA-host interface, the reduction in cell attachment resulted in endophthalmitis and extrusion of the implant. Endophthalmitis is an internal inflammation of the eye, which is a possible complication of B-KPro, arising because of inadequate integration of PMMA with the host corneal tissue allowing penetration of microorganisms. Suggested ways to reduce this drawback include using of daily antibiotics and modifying the B-KPro surface. Using antibiotics is out of the scope of this paper, so only surface modification of PMMA will be discussed.

Surface modifications of PMMA have been studied using two approaches. One of them focuses on improving cell attachment at the PMMA-corneal tissue interface to prevent the penetration of microorganisms into the internal part of the eye. In this context, Patel et al. (2006) studied the region-specific control of cell adhesion by grafting di-amino-PEG onto the surface of PMMA which could be used to improve cell adhesion and spatially control cell attachment onto the PMMA. Recently the stability and biocompatibility of poly (2-hydroxyl methacrylate)-polymethyl methacrylate (PHEMA-PMMA) has been studied and has shown promising optical transparency, flexibility, and good mechanical properties. Nevertheless, its stability and biocompatibility still need further research (Hwang and Kim, 2016). The second approach is to fabricate a PMMA polymer with antibacterial properties. Examples of these antibacterial coatings are titanium dioxide (TiO2) (Riau et al., 2016) and silver nanocluster (Baino et al., 2016). For instance, Salvador-Culla et al. showed that a TiO2 coating on PMMA enhanced keratocyte cell integration and attachment, while at the same time demonstrated antibacterial properties (Salvador-Culla et al., 2016). In another attempt, researchers at the University of California, Irvine (UC Irvine) mimicked the surface of insect wings to make an antibacterial PMMA for using in an artificial cornea (Kowalski, 2016).

In the presence of severe ocular surface inflammation like chemical burns, end-stage Stevens-Johnson syndrome, ocular cicatricial pemphigoid, multiple failed penetrating keratoplasties, and trachoma, the use of the Osteo-Odonto-Keratoprosthesis (OOKP) is an alternative approach. Multiple efforts in using more biologically compatible substances resulted in the introduction of OOKP in 1963 by Strampelli (Han et al., 2015b). In this kind of artificial cornea, an autologous tooth was cut horizontally (OOKP) or longitudinally (MOOKP) to allow an optical cylinder made of PMMA to be introduced inside it. MOOKP is a modified OOKP introduced by Falcinelli et al. (1987). Tibia KPro is another type of biological keratoprosthesis in which a fragment of the autologous tibia was used to allow anatomical retention for an extended period of time. The biological parts of OOKP and MOOKP are known as a “keratoprosthesis skirt” and various studies have suggested that the use of living materials results in lowering the risk of extrusion and infection (Falcinelli et al., 2005). Beside the biological parts of OOKP and MOOKP, two other types of synthetic materials are used in these implants. These are (a) the transparent material, which is used in the optical cylinder, and (b) the adhesive, which is used to bond the optical cylinder to the biological parts. PMMA is a gold standard transparent, water-resistant, and durable material for the optical cylinder. However, as mentioned before, there are ongoing studies toward improving its characteristics. Acrylic bone cement is a standard dental adhesive, which is commonly used, and its suitability has never been seriously challenged. Yet, studies on other dental adhesives like glass ionomer and universal resin cement have been conducted (Weisshuhn et al., 2014; Alarcon et al., 2016).

In comparison with B-KPo, MOOKP is cheaper and does not require a viable donor cornea. However, there are some obstacles and controversial issues related to the use of OOKP and MOOKP. For instance, two fundamental surgical procedures need to be performed to implant the biological keratoprosthesis, and there is a 3-month interval between these procedures. This requirement complicates the surgery and reduces patient satisfaction. As a result, in the last decade, research on OOKP and MOOKP has focused on replacing the biological skirt with a synthetic skirt that enables it to biointegrate with the surrounding corneal tissue. Synthetic skirts have shown better mechanical biocompatibility with the sclera, in addition to more comfortable fabrication and better handling during the surgery. AlphaCor is one of the commercially available artificial corneas with a synthetic skirt. This kind of keratoprosthesis has been studied and developed in the Lion Eye Institute of Western Australia since 1989. AlphaCor is a one-piece device with two concentric regions that made from poly-(2-hydroxyethyl-methacrylate) (PHEMA). The central core of the device is a transparent PHEMA cylinder, and the skirt is a porous PHEMA material to improve biointegration with the surrounding corneal stromal tissue. Nevertheless, the rejection rates of the synthetic skirt is much higher than the biological skirts discussed above (Polisetti et al., 2013). Thus, improving the biointegration of the synthetic skirt is an important objective in recent studies. In this regard, Pino et al. (2008) attempted to increase the bioactivity of various synthetic polymers using biomimetic coatings. They suggested that growing a bioactive apatite layer on the surface of the polymers would provide well *in vivo* biointegration. Another approach to enhancing the bioactivity of the skirt is to modify the surface of the skirt with extracellular matrix proteins like fibronectin, laminin, and collagen (Xie et al., 1997). Moreover, replacing the polymers with bioactive materials like bioglass has also been investigated (Laattala et al., 2011). Accordingly, Huhtinen et al. (2013) replaced the polymeric skirt of the keratoprosthesis with bioactive glass. They claimed that the porous bioglass had a capacity to induce and support tissue ingrowth, resulting in better biointegration. Similar investigations using other innovative materials have been proposed by other research groups. Recently, Tan et al. (2015) assessed the potential of two-dimensional graphene film and 3D graphene foam as a next-generation biomaterial for the synthetic keratoprosthesis skirt.

**Figure 3:**
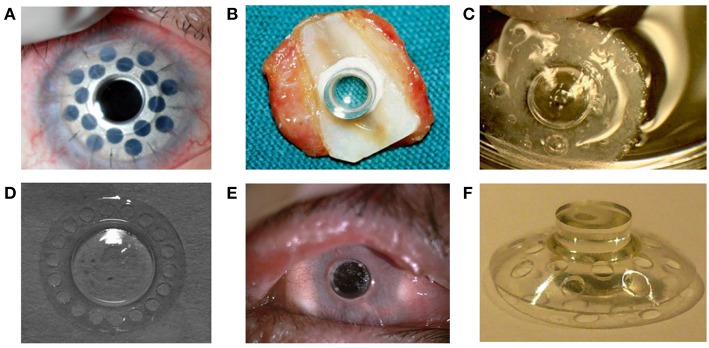
**(A)** Boston keratoprosthesis Type I with titanium back plate. Reprinted with permission from Dohlman et al. (2014). **(B)** Osteodental-acrylic complex with polymethyl methacrylate optical cylinder. **(C)** Both core and skirt are shown in this picture. The skirt is white due to collagen incorporated in the pores. The clear ring between the center and the skirt shows interdigitation between the two components. **(D)** Front profile of the KeraKlear keratoprosthesis demonstrating the 18-hole peripheral design with 4.0 mm central optic. Reprinted with permission from Cortina and De La Cruz (2015). **(E)** MICOF KPro is composed of two parts: a titanium frame and a central PMMA cylinder. Reprinted with permission from Huang et al. (2012). **(F)** MiroCornea UR keratoprosthesis. Reprinted with permission from Duncker et al. (2014).

Considering the advantages and disadvantages of the three commercial keratoprosthesis which have been discussed above, KeraMed Inc. (Sunnyvale, California) has introduced a newer design to address the limitations of previous keratoprostheses while retaining their advantages. KeraKlear Artificial Cornea is a one-piece keratoprosthesis without any need for a donor cornea. Moreover, using only acrylic material allows KeraKlear to be foldable and injectable. However, its implantation is technically challenging, and further investigations are needed to overcome these challenges (Pineda, 2015). Besides, KeraKlear is a new product, and more clinical evaluation is necessary to ensure its safety and efficacy. To summarize the above discussions, the keratoprostheses are compared with two other recently introduced artificial corneas in Table 1 and Figure 3.

**Table 1:** Comparison of major clinically used keratorostheses.

	**Core material**	**Skirt material**	**Surgery stages**	**Further studies on**	**References**
B-KPro	Donor cornea with PMMA	Titanium	1 stages	Improve adhesion between the cornea and the prosthesis	Lee et al., 2017
OOKP	PMMA	Tooth, Tibia	2-3 stages	Replace the biological part	Hille, 2018
Alphacor	PHEMA	Porous PHEMA	2 stages	Improve adhesion between the cornea and the prosthesis	Jirásková et al., 2011
KeraKlear®	Hydrophilic acrylic polymer	Hydrophilic acrylic material	1 stages	Improve adhesion between the cornea and the prosthesis (biointegration)	Pineda, 2015
MICOF^*^	PMMA	Titanium	2 stages	Improve adhesion between the cornea and the prosthesis (biointegration)	Wang et al., 2015; Ma et al., 2017
Miro Cornea®	Hydrophobic acrylic polymer	Hydrophobic acrylic polymer	1 stages	Improve adhesion between the cornea and the prosthesis (biointegration)	Schrage et al., 2014

* *Moscow eye microsurgery complex in Russia*.

## Corneal Wound Healing

**Figure 4:**
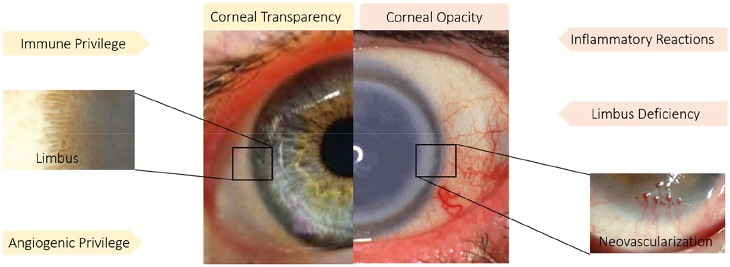
Immune and angiogenic privilege besides limbus structure play a pivotal role in corneal transparency. While inflammatory reaction, neovascularization and limbus deficiency endanger corneal transparency. Reprinted with permission from Ellenberg et al. (2010) and Haagdorens et al. (2016).

Corneal wound healing, like wound healing in general, is a complex and dynamic process which is divided into four phases: the hemostasis, inflammation, cell proliferation, and remodeling phases. In fact, wound healing involves the interaction of various different cell lineages and a choreographed series of cellular events resulting in the replacement of the missing tissue or cellular structures. The cornea has special characteristics, which are important to consider in choosing a treatment strategy for wound healing. The most prominent characteristics of the cornea include its limbus zone, its lack of blood vessels, and its immune privilege (see Figure 4). As a result, current studies in corneal wound healing treatment have focused on a corneal characteristic like an immune response, avoiding angiogenesis and modulating cell signaling. In this section, topics related to angiogenesis and immune privilege, such as blocking immune signaling pathways, exosomes, biopolymers, growth factors, and use of amniotic membrane, will be discussed. New approaches to corneal wound healing related to the limbus zone will be reviewed in the following section (Simpson et al., 2019).

### Corneal Immune Privilege and Avoidance of Angiogenesis

Generally, the transparency and avascularity of the cornea are essential for proper vision. The main factors, which are threatening the corneal clarity are inflammatory reactions, neovascularization, and limbal deficiency. As a result, avoiding angiogenesis, maintaining immune privilege, and supporting the limbus zone are strategies that have been investigated to combat processes that endanger corneal transparency. Immune privilege describes the natural lack of inflammation in the cornea, but when wound healing is taking place, the immune response needs to be modulated and limited. The influx of inflammatory cells causes scar formation and destruction of the limbus zone. Since there is a naturally enhanced production of proangiogenic factors during wound healing (Cursiefen, 2007; Ellenberg et al., 2010), actions need to be taken to restrict the undesirable growth of blood vessels into the cornea. In the following section, recent studies on this issue will be discussed.

#### Blocking Immune Signaling Pathways

Aggressive inflammatory responses following ocular injuries often tend to impair corneal re-epithelization which results in loss of corneal transparency and impairment of vision. Immune responses in cornea have been studied extensively in the context of the role of epithelial cells and immune signaling pathways that are involved. One of the most recognized immune signaling pathways is the inflammasome pathway which is activated in the wound healing response (Schroder and Tschopp, 2010). Recently, Bian et al. described a novel inflammasome signaling pathway which is activated in damaged corneal epithelial cells. They also suggested that blocking this pathway could result in a reduced inflammation which would improve the wound healing and the corneal transparency (Bian et al., 2017).

#### Exosome

Corneal epithelial cells (besides their ability to renew themselves) play a pivotal role in wound healing by carrying out intercellular signaling and communication with stromal cells. Exosomes are cell-derived nanoscale vesicles containing bioactive molecules, which mediate intracellular signaling (Cocucci et al., 2009; Han et al., 2015a). Han et al. have focused on the role of exosomes in corneal wound healing. In their recent study (Han et al., 2017), they characterized corneal epithelial cell-derived exosomes. Their findings indicate the role of exosomes derived from corneal epithelial cells in mediating the intercellular communication between the epithelium and the stroma during corneal wound healing. These exosomes may serve as a therapeutic strategy for the corneal repair.

#### Biopolymers

Common treatments for corneal epithelial defects include artificial tears and lubricants which reduce the mechanical stress and inflammatory cytokines. Inhibiting the inflammatory responses may result in the acceleration of corneal wound healing (Zarrintaj et al., 2018d). Inasmuch as biopolymers have shown promising results in tissue engineering and wound healing, their deployment in corneal wound healing has attracted lots of attention (Gholipourmalekabadi et al., 2018; Zarrintaj et al., 2018c). Chitosan, hyaluronic acid, silk fibroin, and polyarginine are among the most studied biopolymers for corneal wound healing. Chitosan and its derivatives have a large number of applications in the human body in various forms, such as scaffolds, drug carriers, and wound dressings (Dai et al., 2011; St. Denis et al., 2012; Oryan and Sahvieh, 2017; Sigroha and Khatkar, 2017). In regard to corneal wound healing, Cui et al. (2017) recently have reported a mechanism by which chitosan could promote corneal wound healing. Their findings showed that the chitosan-stimulated epithelial wound healing was partially mediated through the activation of the extracellular signal-regulated kinase (ERK) pathway. ERK signaling plays a pivotal role in cell proliferation, migration, and differentiation. Chitosan and its derivatives have also been studied as a drug carrier in ocular wound healing (Schmidl et al., 2017; Rahmati and Mozafari, 2019a). Fischak et al. (2017) studied the effects of chitosan-N-acetylcysteine (C-NAC), a new biopolymer, on corneal wound healing. The results showed a faster wound healing due to the specific chemical and biological properties of C-NAC. Other chitosan compositions which have been investigated for corneal wound healing are chitosan-based hydrogels. These hydrogels may either serve as drug carriers (Tsai et al., 2016) or as tissue adhesive materials for hemostasis or wound healing (Deng et al., 2010; Lih et al., 2012; Wicklein et al., 2019).

Hyaluronic acid is one of the abundant polysaccharides in the human body and plays a significant role in corneal wound healing. *In vitro* and *in vivo* studies have confirmed the ability of hyaluronic acid to promote wound healing (Neuman et al., 2015). Zhong et al. (2016) studied the mechanism by which exogenous hyaluronic acid promotes corneal wound healing. They studied the expression level of cytokines like Cluster of differentiation (CD44), interferon (IFN), interleukin 1 beta (IL-1β), and matrix metallopeptidase 9 (MMP-9). Hyaluronic acid down-regulates the expression of inflammatory cytokines and up-regulates the expression of anti-inflammatory cytokines associated with the tissue repair and healing. Though, despite the confirmed effects of hyaluronic acid on promoting corneal wound healing, Gronkiewicz et al. (2017) reported that the topical addition of hyaluronic acid, in combination with standard medical management of corneal ulcers, did not accelerate wound healing.

Fibroin is an insoluble protein derived from the fibers of silk. Hydrophobic domains in the primary sequence of amino acids in fibroin generally result in this protein adopting a β-sheet structure (Vepari and Kaplan, 2007; Mohammadi et al., 2017; Rahmati and Mozafari, 2018). Lui et al. studied fibroin as a 2D and 3D-scaffold for corneal stromal engineering applications (Liu et al., 2012) and as a carrier for exogenous application of corneal epithelial cell sheets (Lawrence et al., 2009). Recently, Abdel-Naby et al. (2017) evaluated the influence of fibroin on epithelial cell migration, proliferation, and adhesion. Their results indicated that fibroin might directly enhance wound healing by both stimulating epithelial proliferation and positively impacting the cell migration rate.

Polyarginine is a short cationic polypeptide, which can translocate through cell membranes; as a result, it has attracted much attention as a drug carrier. Some studies have shown that the presence of guanidinium moieties in the backbone of polyarginine, which interacts with anionic groups on the cell membrane through hydrogen bonds and hydrophobic forces, results in the cell-penetrating property of polyarginine (Takechi et al., 2012). Studies of polyarginine as a nanocarrier have significantly increased in recent years, and it has emerged as a new strategy to accelerate wound healing (Gonzalez-Paredes et al., 2017). Reimondez-Troitiño et al. (2016) designed and evaluated polyarginine nanocapsules to improve corneal wound healing. Their findings showed that polyarginine had an intrinsic capacity to promote corneal wound healing through the transforming growth factor beta /SMAD (TGF-β/SMAD) signaling pathway.

#### Amniotic Membrane (AM)

Both fresh and preserved human amniotic membranes have been investigated as naturally occurring biomaterials in tissue reconstruction, especially for the ocular surface. AM is one of the thickest basement membranes that exists in the human body, with the ability to promote epithelial cell healing, besides inhibiting fibroblast proliferation and myofibroblast differentiation. In addition, it contains several anti-angiogenic, anti-inflammatory, and neurotrophic factors (Ramachandran et al., 2019). At present, AM transplantation has been used for various indications including repairing persistent epithelial defects and treating corneal ulceration, limbal stem cell deficiency, acute Stevens Johnson Syndrome (SJS), chemical and thermal burns, infectious keratitis, and after refractive surgery (St. Denis et al., 2012; Manolova et al., 2017; Prabhasawat, 2017; Westekemper et al., 2017). Despite the fact that during the last decades, AM transplantation has been a gold standard for the treatment of a variety of ocular surface diseases, AM transplantation still has several disadvantages. Therefore, multiple efforts have been made to address these downsides. One of the disadvantages is related to the surgical procedure that has several problems. The precise conditions, under which the AM is prepared, affects its biomedical applications (Islam et al., 2018). Wu et al. (2017) analyzed the effect of two different methods of preparations of AM on human corneal epithelial cell (HCEC) viability, migration, and proliferation *in vitro*. Their study showed that biochemical factors (Keratinocyte growth factor (KGF), Fibroblast growth factor-basic (FGFb), Hepatocyte growth factor (HGF), and TGF-β1) released from the AM preparation had a complex, possibly non-linear effects, on HCECs. In another study Ogawa et al. identified an active matrix component [Heavy chain-hyaluronan/pentraxin3 (HC-HA/PTX3)] that was shown to exert the anti-inflammatory and anti-scarring effects of AM. Their studies revealed that subcutaneous and subconjunctival injection of HC-HA/PTX3 might be a novel approach in the treatment of ocular disease (He et al., 2017a; Ogawa et al., 2017). Recently, this group developed eye drops containing morselized and cryopreserved AM, and also an umbilical cord preparation to understand their therapeutic potential in promoting corneal re-epithelization and restoring the regularity of the corneal surface (Tighe et al., 2017).

#### Pharmaceutical Agents

Pathological angiogenesis that occurs in the proliferative phase of corneal wound healing leads to a reduced corneal transparency and loss of vision via lipid deposition and scar formation. Neovascularization is closely related to the angiogenic signaling pathway, which is initiated by the infiltration into the cornea of large numbers of neutrophils and macrophages. Several studies have shown the potential benefit of using anti-angiogenic agents to inhibit corneal neovascularization. Different methods have been suggested to inhibit the development of corneal neovascularization like genetic ablation of the chemokine receptor CCR2 (Ambati et al., 2003) and to control pro-angiogenic factors (Baradaran-Rafii et al., 2017). Zerumbone is a cyclic terpene which is isolated from the rhizomes of wild ginger. Presently, zerumbone has been extensively studied for its antitumor, anti-inflammatory, antimicrobial, and anti-angiogenic activities (Rahman et al., 2014). Kim et al. (2017b) recently examined the effects of zerumbone on chemokine-related macrophage infiltration in the corneal wound healing process. Their results indicated that zerumbone prevented angiogenesis and fibrosis through the inhibition of inflammatory cells activation.

Anti-inflammatory agents like corticosteroids have long been topically applied for the treatment of ocular inflammation. However, they show a low therapeutic efficacy in the treatment of neovascularization, because of their poor corneal permeability, and lack of bioavailability (Mozafari, 2014). Various drug delivery systems like viscous solutions, nanoparticles, carbon nanotubes, micelles, liposomes, and hydrogels have been proposed to overcome the aforementioned problems (Karimi et al., 2015a,b, 2016a,b,c; Weng et al., 2017). Recently, Nagai et al. (2017) designed a new type of solid nanoparticles based on zirconia beads containing dexamethasone, to improve drug permeability through the cornea. These solid nanoparticles facilitated topical passage of dexamethasone through the barriers of the eye. With the goal of combining the prevention of neovascularization and reduction of inflammation, Huang et al. (2017) designed a supramolecular hydrogel for co-delivery of dexamethasone sodium phosphate and Avastin® (an antiangiogenic agent). The supramolecular hydrogel was composed of MPEEG-PCL micelles and α-cyclodextrin (an oligosaccharide cage). The *in vivo* studies showed that this supramolecular hydrogel significantly attenuated the inflammatory response and inhibited neovascularization through downregulation of the vascular endothelial growth factor (VEGF), CD31, and alpha-smooth muscle actin (α-SMA) expression.

Natural tears have trophic effects on epithelial cells because they contain vitamins, immunoglobulins, proteins, growth factors, and electrolytes (Grigoryeva et al., 2013). “Autologous serum eye drops” contain several essential nutrients like growth factors, vitamins, cytokines, proteins, and lipids that may assist in corneal re-epithelization. In addition to autologous serum, bandage-type contact lenses have been studied for repairing the corneal epithelial defect. Bandage contact lenses prevent the mechanical tension associated with blinking, therefore reducing necrosis and desquamation of the corneal epithelium (Ho and Mathews, 2017). Several studies have been done to combine the therapeutic effects of autologous serum and bandage contact lenses (Schrader et al., 2006; Choi and Chung, 2011). In a recent study, Wang et al. (2017) described the therapeutic outcomes of a combination of a topical 20% autologous serum and a silicone hydrogel contact lens in 12 patients during a 3-month follow-up period. All patients suffering from post-infection corneal epithelial defects were successfully treated; “corneal melting” during acute disease was successfully prevented. Studies have shown that breast milk performs similarly to autologous serum and natural tears. Thus, breast milk may accelerate epithelial wound healing because it contains anti-infection agents and growth factors. In one study, Asena et al. (2017) compared human breast milk with autologous serum and artificial tears in corneal epithelial wound healing. Their results showed that the presence of growth factors like TGF-β, insulin-like growth factor-1 (IGF-1), lipids, and vitamins in breast milk played important roles in epithelial and stromal wound healing in the cornea.

**Figure 5:**
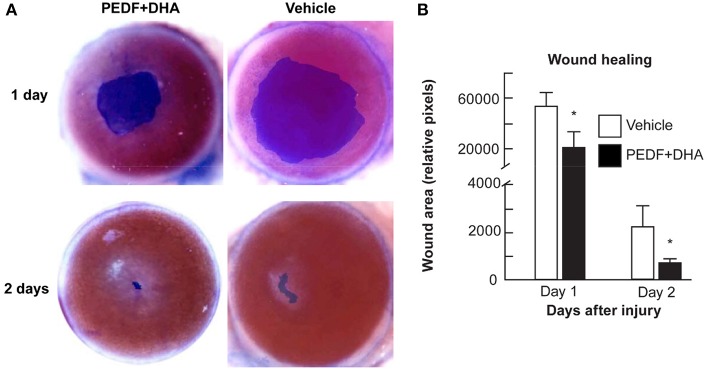
Effect of PEDF+DHA treatment on wound healing in diabetic corneas. The right eyes of 16 mice with hyperglycemia for 10 weeks were injured and divided randomly into two groups and treated for 1 or 2 days with PEDF+DHA or vehicle. **(A)** The wounded corneas were stained with 0.5% methylene blue and photographed with a surgical microscopy through an attached digital camera. **(B)** Wounded area. Data is expressed as mean ± SD (^*^p < 0.05, *n* = 4 mice/group). Reprinted with permission from He et al. (2017b).

A novel therapeutic approach, described by Bazen and his colleague at Louisiana University, employed a neurotrophic and anti-angiogenic factors (pigment epithelium-derived factor [PEDF]) combined with an essential fatty acid (docosahexaenoic acid, DHA] stimulated nerve regeneration in diabetic keratopathy (Bazan et al., 2014; He et al., 2015). In their recent study, they evaluated the therapeutic effects of topical application of PEDF and DHA over 2 weeks on the regeneration of the corneal sensory nerve in both wounded and unwounded diabetic corneas. They proposed some possible mechanisms for their results including neuroprotective and anti-oxidant actions of the PEDF+DHA, neurotrophic function, and the anti-inflammatory activity of this treatment. Figure 5 shows promoted wound healing on days one and two after injuries in the PEDF+DHA treated corneas (He et al., 2017b).

Another pharmaceutical agent, which has recently gained much attention, is a new type of extracellular matrix agent called ReGeneraTingAgent or RGTA (Arvola et al., 2016; Chappelet et al., 2017; Robciuc et al., 2018). This approach consists of engineered polymers designed to protect and replace cellular signaling proteins of the extracellular matrix (ECM), such as heparan sulfate. This unique property encourages the reconstruction of the ECM; therefore facilitating tissue wound healing (Barritault et al., 2017). Recently Gumus et al. (2017) investigated the topical effects of a biodegradable nanopolymer (alpha 1-6 polycarboxymethyl-sulfate) to mimic heparan sulfate in order to accelerate corneal re-epithelization and stromal healing, after epi-off corneal cross-linking technique. In fact, RGTAs were engineered to bind to heparan sulfate binding sites on proteins of the ECM. These agents are large enough to bridge between neighboring matrix proteins and recreate a cellular microenvironment and a microniche, where cells can respond appropriately to the cascade of signals involved in the wound healing process.

**Figure 6:**
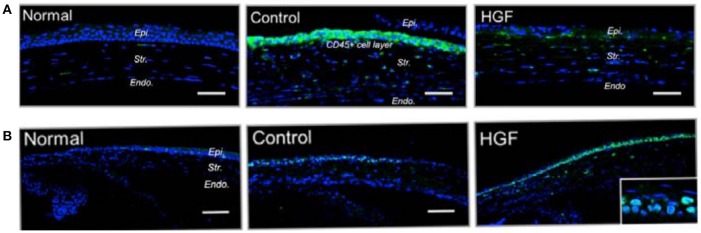
Mechanical injury was induced in murine corneas by scraping the epithelium and either topical recombinant hepatocyte growth factor (HGF) was applied twice daily. Normal corneas without injury or injured corneas receiving MSA served as controls. Corneas were harvested at day 3 after injury. **(A)** Representative immunofluorescent images of sections of mouse cornea stained for Ki-67 (green) showing proliferating cells (scale bar, 100 mm). **(B)** Representative immunofluorescence images of corneal cross sections showing higher expression of CD45 (green) in MSA-treated controls, compared to HGF-treated eyes (scale bar, 50 mm). Reprinted with permission from Omoto et al. (2017).

Many investigations have emphasized the role of several growth factors like platelet-derived growth factor (PDGF), VEGF, TGF-β, HGF, and tumor necrosis factor-beta (TNF-β) in the wound healing process. Among these growth factors, PDGF, TGF-β, and HGF played a pivotal role in modulating cell proliferation and myofibroblast differentiation. There is extensive literature available concerning the role of growth factors in corneal wound healing (Gallego-Muñoz et al., 2017; Sriram et al., 2017). In fact, a better understanding of the role of growth factors in corneal wound healing would likely lead to the development of new treatments. In one of the corresponding studies, Omoto et al. (2017) reported the effects of topical administration of HGF on inflammation of corneal epithelial cells. Their results demonstrated that topical application of HGF promoted corneal epithelial cell proliferation which was revealed by higher expression of the Ki-67 and p63 proliferation markers in HGF-treated mice. In addition, HGF treatment reversed the anti-proliferative effect of IL-1β *in vitro*, indicating that HGF actively suppressed the inflammatory environment in the corneal epithelium. On the other hand, HGF significantly reduced the infiltration of CD45+ inflammatory cells in the cornea (see Figure 6).

### Limbus Zone

**Figure 7:**
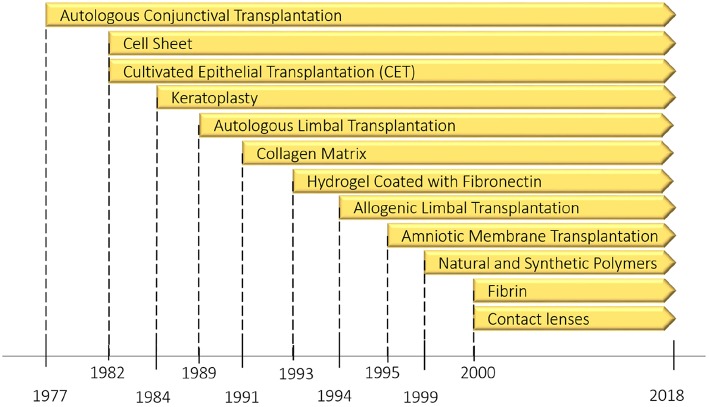
The evolution pathway of ocular surface reconstruction investigations which started with autologous conjunctival transplantation in a patient with bilateral alkali burn in 1977 and have been continued with other methods specially limbus regeneration over the past four decades. Partially reprinted with permission from Nakamura et al. (2016).

The limbus of the cornea forms a border between the corneal and conjunctival epithelium and its limbal stem cells (LSCs) are essential in the maintenance and repair of the adult cornea as they support the repair and regeneration of corneal epithelial tissue. In fact, this undulated limbal region is considered to be a niche for LSCs, which play a critical role in the corneal wound healing process. In limbal tissue engineering, like the other tissues, three factors affect the regeneration of injured tissues: cell availability, suitable and biocompatible scaffolds, and the presence of growth factors. In corneal disorders, depletion or the absence of LSCs results in impairment of the corneal wound healing process. Regarding tissue engineering, there are three approaches to remedy this deficiency. The first approach is cell transplantation using the desired cell population obtained in tissue culture. This approach has been considered as a type of corneal transplantation that was briefly discussed in the previous section. The second approach is cell transplantation using cells such as corneal epithelial, where stem cells are dissociated, cultivated on a supportive matrix (biosynthetic scaffold) like an amniotic membrane, fibrin gel or polymers, and then injected into the desired location in the cornea. The main problem in the last approach is the lack of stem cell enrichment because the stem cells which are used for transplantation contain heterogeneous cell populations. This deficiency might result in a graft failure (Rajendran et al., 2017). As an alternative solution, a few groups have recently focused on a new approach by mimicking the LSC microenvironment (niche) in which the LSCs live. In this section, new progress and emerging alternatives related to the second and third approaches will be discussed. Moreover, in order to get a broad overview of the subject, Figure 7 covers the evolutionary pathway of limbus regeneration over the past four decades.

#### Cell Transplantation

Using the bioengineered cornea is an alternative approach to addressing the aforementioned restrictions of corneal transplantation and artificial corneas. In most articles, tissue engineering of the outermost layer of the eye is known as ocular surface regeneration. In this approach, the optical and biomechanical characteristics of the tissue-engineered cornea are essential. For instance, tissue engineered cornea must be transparent and withstand about 10–20 mm Hg of intraocular pressure. Therefore, biomaterial selection to produce appropriate scaffolds is an ongoing subject of investigation due to the emergence of tissue engineering. The main approaches in corneal tissue engineering to repair corneal defects can be divided into full thickness, stromal, epithelial, and endothelial types of regeneration. All of them involve the aid of a scaffold in combination with different cell types. Recent advances in corneal tissue engineering have been reviewed by Ghezzi et al. (2015). Meanwhile, the most studied biomaterials are amniotic membrane, collagen matrix, hydrogels, and other natural and synthetic polymers. After choosing the necessary materials, the physical, chemical, and biochemical modification of these materials play a pivotal role in the results. In this section, we have tried to review these modifications (Ismail et al., 2019; Wijnholds, 2019).

##### Amniotic membrane (AM)

**Figure 8:**
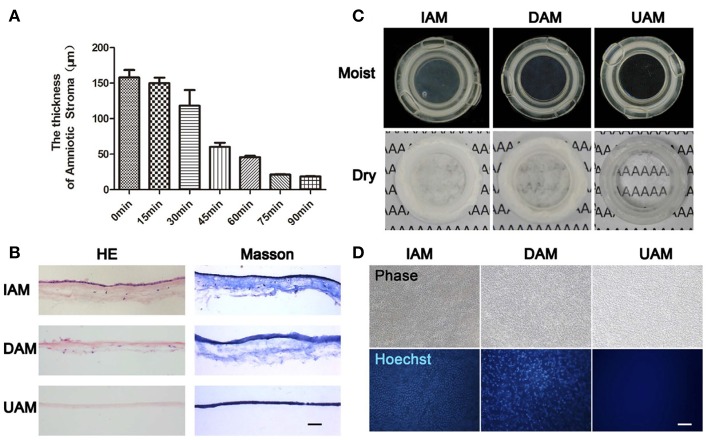
The characteristics of ultra-thin amniotic membrane. **(A)** The thickness change of amniotic membrane after digestion with collagenase type IV for different time durations. **(B)** HandE and Masson trichrome staining of IAM, DAM, and UAM tissues (Bar: 100 μ m). **(C)** Macroscopic views of IAM, DAM, and UAM were evaluated by photography scanning in moist form and light microscope in freeze dry form. **(D)** Hoechst whole mount staining of IAM, DAM, and UAM (Bar: 100 μ m). Reprinted with permission from Zakaria et al. (2014).

Due to the specific characteristics which were described in detail in the last section, the gold standard substrate for the *ex vivo* expansion of LSCs remains AM. In recent years, different studies have focused on AM as a biological carrier in *ex vivo* reconstruction and transplantation of tissue engineered corneal epithelium (Sabater and Perez, 2017). Various aspects of this strategy have been investigated. One of these aspects is the relative transparency of the AM. In fact, AM features lower clarity than human cornea, and this lack of transparency restricts the use of AM as a carrier in corneal epithelial tissue engineering. Zhang et al. (2016) proposed a thinning protocol for the generation of ultra-thin amniotic membrane. The prepared AM was transparent and composed of a compact transparent layer which made it an ideal carrier for the construction of tissue engineered corneal epithelium (see Figure 8). Another challenge in using the AM is its potential to transmit infectious disease. Decellularization processes have been applied to various types of tissue for laboratory investigations and clinical applications. The most important aspects of any tissue decellularization protocol are to eliminate the risk of immune rejection and disease transmission, while at the same time retaining the underlying ECM structure. In this regard, Figueiredo et al. (2017) studied the potential use of decellularized human amniotic membrane for the *ex vivo* expansion of LSCs. They also evaluated, using gamma irradiation as a final sterilization step, the potential minimization of risk regarding disease transmission. Their results showed that *ex vivo* expansion of LSCs using an explant culture system occurred at a faster rate on decellularized human AM in comparison with fresh human AM. Human amniotic membrane is in clinical trials for corneal wound healing (Zakaria et al., 2014).

##### Fibrin gel

Fibrin was first introduced for wound healing applications in the form of tissue glue or engineered sheets. Fibrin is a natural protein involved in blood coagulation and, because of its biocompatibility, biodegradability, and its potential in wound healing, has attracted much interest in corneal wound healing (Ronfard et al., 1991; Pellegrini et al., 1999). In corneal surface reconstruction fibrin has been used as a glue for tissue adhesion or as a fibrin gel acting as a carrier of LSCs. The first studies on fibrin gel were carried out in early 2000s by Rama et al. (2001), Ronfard et al. (2000), Duchesne et al. (2001), and Han et al. (2002). Among these groups, Rama et al. published a long term follow-up (up to 10 years) in 2010, and their results showed that the renewal of corneal epithelium was attained in 76.6% of 112 patient eyes (Rama et al., 2010). Rama et al. also studied the 3T3 cell line cultured on fibrin matrix as a feeder-layer which could supply metabolites to the LSCs. Recently, Lužnik et al. (2017) have studied the possibility of omitting the feeder-layer to achieve the xeno-free scaffold. Fibrin gel is in the clinical trial to investigate the cultivated oral mucosal epithelial cell sheet transplantation (COMET) of substrate-free cell sheets on reconstructing the ocular surface. In the study, fibrin-coated dishes with proteins inhibitor was used to adjust the degree of fibrin degradation (Hirayama et al., 2012). Nevertheless, studies on fibrin as a matrix have been overshadowed by other types of natural and synthetic polymers.

##### Natural and synthetic polymers

Natural and synthetic polymers have been the subject of several studies aiming to find a suitable carrier for the transfer of stem cells. In this regard, various polymers like hydrogels, self-assembling peptide nanofibers, collagen matrix, conductive polymers, and thermosensitive polymers have been studied (Deng et al., 2010; Zarrintaj et al., 2018a,b). Mechanical strength and transparency are the most important considerations in order to reconstruct the injured cornea (Fagerholm et al., 2010; Mozafari et al., 2019; Wicklein et al., 2019). In Table 2 pioneering studies on polymers as a substrate for corneal tissue engineering are summarized.

**Table 2:** Natural and synthetic polymers used for corneal wound healing.

**Carrier**	**Novelty**	**Target tissue**	**results**	**References**
Gelatin/ascorbic acid (AA) cryogel	Using antioxidant molecule-mediated structure	Corneal stroma tissue engineering	Low-to-moderate AA loading demonstrated better capability to enhance tissue matrix regeneration and transparency maintenance in animal model.	Luo et al., 2018
Plastic compressed collagen gel/electrospun poly(lactic-co-glycolide) (PLGA) mats	Laser-perforating sandwich-like hybrid construct	Corneal epithelial and stroma tissue engineering	Co-culture of two kinds of cells for corneal tissue reconstruction	Kong et al., 2017
Aligned silk membrane	Multi-layered silk membrane with neuropeptide substrate	Corneal stroma tissue engineering	Differentiating periodontal ligament stem cells (PDLSCs) toward keratocytes	Chen et al., 2017
Silk/polyurethane hybrid nanofibrous	Using conjunctiva derived mesenchymal stem cell(CJMSCs) as a new source for differentiation	Corneal epithelial tissue engineering	Interconnected pore to accelerate nutrient diffusion with sufficient mechanical properties	Soleimanifar et al., 2017
Thermosensitive chitosan-gelatin hydrogel	Human stromal cell-derived factor-1 alpha (SDF-1 alpha) loaded	Corneal epithelial tissue engineering	Exogenous SDF-1 alpha promotes corneal epithelium reconstruction through increase local expression of other growth factor	Tang et al., 2017
Collagen type-I coated poly(lactic-co-glycolic acid) film	Using hybrid graft	Corneal endothelial tissue engineering	Limiting the probability of non-specific interaction between the construct and the biological environment	Kim et al., 2017a
Hyaluronic acid/pluronic hydrogel	Injectable hydrogel with porcine platelet rich plasma(P-PRP)	Corneal endothelial tissue engineering	Limiting hydrogel-induced cell death	Lin et al., 2017
Silk fibroin	Developing artificial endothelial graft	Corneal endothelial tissue engineering	Appropriate biological properties beside mechanical properties that allowed its use in a Descemet membrane endothelial keratoplasty	Vázquez et al., 2017
Poly(glycerol sebacate)PGS-poly (ε-caprolactone) PCL nanofibrous	Elastomeric biodegradable scaffold	Corneal endothelial tissue engineering	Semi-transparent and highly elastic aligned nanofibrous PGS-PCL blended scaffold	Salehi et al., 2017
Sequential hybrid crosslinking gelatin methacrylate	Hydrogel patterning with Nanoscale resolution	Corneal endothelial tissue engineering	Increased mechanical strength, transparent and provide adequate nutrient transport	Rizwan et al., 2017
Gelatin microcarriers Functionalized with oxidized hyaluronic acid	Using cell-containing microcarriers	Corneal stromal tissue engineering	Microcarriers well tolerated and can be degraded by endogenous enzymes following intracameral implantation	Lai and Ma, 2017
Short collagen-like peptides conjugated to polyethylene glycol	Using synthetic and customizable analogs	Corneal endothelial tissue engineering	Promoting corneal regeneration through stimulation of extracellular vesicle production by endogenous host cells that migrate into the scaffold	Jangamreddy et al., 2018

##### Contact lens

Despite the promising clinical outcomes of AM and fibrin gel, the study of other transplantation procedures has been continued. Among them, the carrier-free transfer method (cell sheets), and the use of a contact lens have gained more attention as they reduce the risk of xenobiotic infections and side effects from using non-Food and Drug Administration (FDA) approved biomaterials. Girolamo's group developed a novel autologous technique by using an FDA-approved soft contact lens as a carrier and bandage to protect the eye during LSC transplantation, thereby promoting corneal repair and regeneration (Di Girolamo et al., 2009). Their cell-laden siloxane-hydrogel contact lens was successful in reconstituting a healthy ocular surface in 16 patients with limbal stem cell deficiency (LSCD) (Bobba et al., 2015). Studies by several groups have reported attempts to modify the tissue adherence to the carrier, and to vary the stem cell type in order to improve the efficiency of the explant culture system. Tóth et al. (2017) tested several methods for affixing the cells to prevent them from “floating off” the contact lens. Their results showed that in comparison with cyanoacrylate, silicon, and fibrin glue, suturing was the most efficient method to improve tissue adherence. In treating bilateral LSCD, the oral mucosal epithelium was shown to be a suitable autologous stem cell source. Accordingly, Zsebik et al. (2017) cultured human oral mucosal epithelium as a source for cell expansion on a lotrafilcon (a contact lens). They claimed that they established a xenobiotic-free culture system from human oral mucosal explants on the contact lens surface.

##### Cell sheet

Over the past 30 years, conventional tissue engineering approaches (like the use of biodegradable scaffolds and/or injection of isolated cell suspensions) have been successful in the regeneration of different tissues (Koizumi and Okumura, 2019). However, these tissue engineering approaches still face some obstacles when applied for reconstruction of the ocular surface. There is a significant risk of infection when biological materials are employed, the attachment to the replacement site can be insufficient and can lead to degradation and loss of optical transparency. Sheet-like cell assemblies were introduced as a method to address these limitations (Green et al., 1979; Fagerholm et al., 2014). In this cell sheet technology, the desired cells are grown on a particular cell culture surface which allows reversal of cell adhesion, so an intact cell sheet can be transplanted to the host tissue without using scaffolds. In cell sheet technology several external stimuli have been applied to facilitate cell detachment like specific enzymes, temperature variation, magnetic force, electrochemical polarization, pH variation, polyelectrolytes, and illumination (Owaki et al., 2014). The cell sheet technique needs further study to become standardized before it can be used in routine clinical practices. One of the obstacles in this method is to find the optimal preservation medium to maintain the viability of the reconstructed tissue. Recently, Katori et al. (2016) introduced a new preservation medium for cell sheets (containing the antioxidant ebselen) derived from human corneal tissue and human oral mucosal epithelium. Another attempt to improve this technique was reported by Syed-Picard et al. (2018). They designed a highly organized structure with aligned microgrooves which directed parallel cell alignment, and allowed matrix organization, similar to that of native corneal stromal lamella (Buznyk et al., 2015). As a result, after transplantation of the engineered corneal tissues, the tissue sheets were incorporated into surrounding tissues and became transparent. A research group led by Okano at Tokyo University has studied the production of cell sheets from the corneal epithelium. They used a temperature responsive polymer to generate multilayered corneal epithelial sheets (Nishida et al., 2004a). Clinical results in four patients with the use of cultured autologous oral mucosal epithelial cell sheets showed that corneal transparency was restored, along with remarkable improvements in postoperative visual acuity (Nishida et al., 2004b). Recently they tried to optimize the cell sheet fabrication process in order to improve cell sheet quality and decrease risk of biological contamination. They developed a “cell cartridge” which acted as a closed culture system for regenerative medicine (Kobayashi et al., 2013; Nakajima et al., 2015). Self-lifting analogous tissue equivalent (SLATE) is a bio-fabricated, scaffold-free system which has been recently used in corneal tissue engineering. Peptide amphiphile used as a surface template provides a platform to control the structural, mechanical, and biofunctional properties of the SLATE to replace damaged corneal tissue. In this study, SLATEs were implanted in a rabbit corneal. After a 9-months follow-up, SLATE was well integrated with surrounding hot tissue without a sign of rejection and provoking inflammation (Gouveia et al., 2017).

#### Artificial Limbus

**Figure 9:**
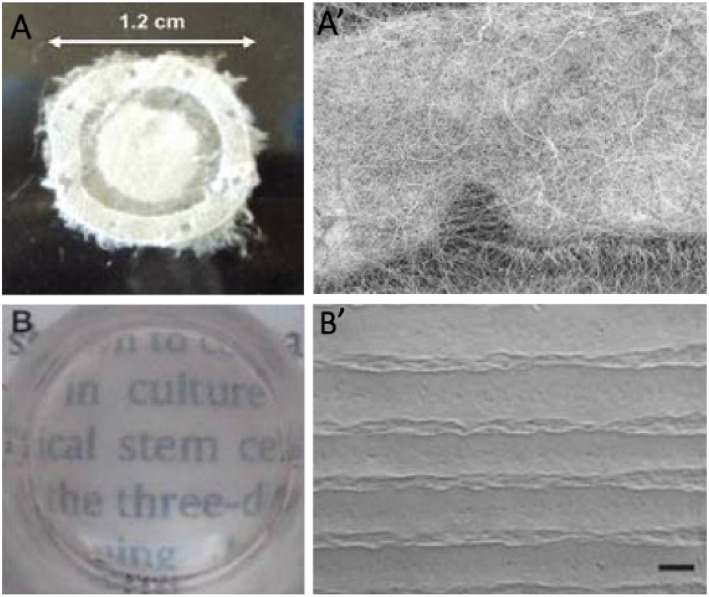
**(A)** Detail of electrospun outer ring with 1.2 cm of diameter, **(A′)** SEM micrograph of a section of the electrospun scaffold showing a horseshoe electrospun micropocket. **(B)** Stability and transparency of cultured RAFT (Ortega et al., 2013b). **(B′)** SEM image of bioengineered limbal crypts on the RAFT surface. Scale bar 200 μm. Reprinted with permission from Levis and Daniels (2016).

“Form follows function” is a well-known concept accepted as a principle in the field of modernist architecture; although it is also universally observed throughout the nature. According to this principle, the shape of an object should be predominantly based upon its intended function or purpose (Griffith and Harkin, 2014; Ghaffari et al., 2016). The application of this principle in the engineering of limbus tissue can be translated into mimicking the three-dimensional morphological structure of tissues in order to provide a specific niche to support stem cells. In the last decade, an emphasis on the importance of architectural features in tissues is a sign that attention is being paid to this principle. Meanwhile, the study of the stem cell microenvironment (niche) and its highly regulated and specific structure has attracted more attention (Rahmati and Mozafari, 2019b). Stem cells are defined by their ability to self-renew and to participate in the regeneration of damaged tissues, and their niche exhibits distinct anatomical and biochemical features in comparison with their surrounding tissue. On the other hand, some studies have shown niche morphology was altered in limbal stem cell deficiency, caused by disease or increased age (Zheng and Xu, 2008; Lagali et al., 2013). Researchers have attempted to characterize and quantify the stem cell niche by various visualization methods, and to utilize these data to fabricate artificial niches for stem cells. Several techniques are now under investigation in order to mimic the niche structure for corneal repair (Ortega et al., 2012). In a recent study, Claeyssens et al. focused on the characterization and evaluation of the impact of the “Palisades of Vogt” structure on the function of limbal stem cells. They combined micro-stereolithography and electrospinning to fabricate PLGA rings containing microfabricated pockets. In this study, they reported the physical and protective properties of the niche. Their results showed that micropockets of the PLGA ring played a pivotal role in cell migration and directionality (Ortega et al., 2013b, 2014a,b). They also studied the fabrication of polyethylene glycol diacrylate rings containing microfeatures, which were modified by biotinylated fibronectin. In their study, the use of ECM proteins stimulated limbal cell outgrowth and migration (Ortega et al., 2013a). Levis group at UCL Institute of Ophthalmology have developed a method to create bioengineered limbal crypts with a functional 3D niche architecture (Levis et al., 2013). This novel process for production was referred to as “Real Architecture For 3D Tissue” or RAFT™. Figure 9 illustrates two kinds of cultured RAFT limbal crypts. This new 3D cell culture system uses physiologically relevant concentrations of collagen to create the most natural environment for cells. The custom molded micro-ridges on the surface of the thin collagen resembles the dimensions of the stromal crypts in the human limbus. They also studied the effect of the 3D topography and ECM markers on the human limbal epithelium. The bioengineered limbal crypts expressed putative limbal epithelial stem cell markers like ΔNp63a and BMi1 and also produced basement membrane proteins like laminin-β1 and laminin-γ3 (Massie et al., 2014; Levis and Daniels, 2016).

## Conclusion and Future Outlook

From the earliest concepts such as replacement of the opaque cornea, to cornea wound healing and regeneration, ophthalmologists, and material scientists worldwide have faced a variety of challenges. Advances in visualization techniques and histology have made significant progress in the fundamental understanding of cornea structure and its microenvironment. Due to this valuable information and nanotechnology advances, therapeutic strategies in devastating corneal diseases have turned from corneal replacement into corneal wound healing and regeneration (Kargozar and Mozafari, 2018). Consequently, studies on the limbus zone and immune and angiogenic privilege have attracted more attention. In addition, the exploration of cell signaling in the natural process of wound healing and the attempts to mimic this process have opened new horizons in corneal disease treatment.

Most of the suggested treatments have shown promising results for wound healing at the ocular surface, and entire thickness dystrophies were neglected. While, in order to reduce transplantation of a donor cornea, tissue engineering of the whole thickness of the cornea must be considered. Corneal stromal and endothelium tissue engineering have recently shown noticeable progress (Matthyssen et al., 2018). However, more focus is needed on biomimetic strategies, like employing a combination of cell signaling agents with tissue engineering. Rho-kinase (ROCK) inhibitor is a serine/threonine protein kinase that participates in regulating cell signaling pathway. Recently ROCK has been introduced as an innovative therapeutic agent for corneal endothelial dystrophy (Han et al., 2018). The compilation of these approaches can be a promising method for visual rehabilitation in patients suffering from corneal dystrophies.

Until now, most studies have worked on introducing new materials and biochemical approaches in cornea wound healing and regeneration; while paying attention to physical properties of these approaches might be a leap in this area. For instance Long et al. have tried to use a cross-linking agent in collagen membrane to regulate collagen fibril spacing and therefore improve optical clarity of collagen and increase permeability of neurites (Long et al., 2018). Hence, advances in visualization techniques will help to improve corneal physical structure identification that, in combination with material science, will lead to new sights in the typical treatment approaches. Slip-lamp biomacroscopy, optical coherence tomography (OCT), *in vivo* confocal fluorescence microscopy, and full-field optical microscopy are part of visualization techniques which help to quantify corneal architecture (Grieve et al., 2015; Werkmeister et al., 2017). According to previous studies, investigation on visualization methods would boost corneal medical treatments.

Considering the remarkable role of stem cells in tissue regeneration, a large part of future studies is expected to focus on the deployment of stem cells on cornea wound healing and regeneration (Rahmati et al., 2018). Several studies have been done to isolate and characterize multipotent stem cells from various tissues in order to use their great potential in regenerative medicine. Bone marrow-derived mesenchymal stem cells (Islam et al., 2018), human umbilical cord mesenchymal stem cells (Yamashita et al., 2018), postnatal periodontal ligament (Yam et al., 2018), and limbal stem cells (Inatomi et al., 2018; Sasamoto et al., 2018) are recently studied stem cells sources in corneal wound healing and regeneration. Saghizadeh et al. (2017) have recently reviewed all major stem cells usage in corneal wound healing. On the other hand, developing innovative methods to produce 3D tissue-like architecture has allowed mimicking the microarchitecture and physiology of the native cornea. In this regards 3D microfabrication methods are promising approaches in designing cornea substitutes (Prina et al., 2017; Ludwig et al., 2018). Among additive manufacturing methods, study on bioprinting and the development of bioinks provides great promise regarding the fabrication of human corneal substitutes that mimic the structure of native corneal tissues (Isaacson et al., 2018; Sorkio et al., 2018).

## Author Contributions

MoM and RA wrote the first draft. SO and MG wrote the first draft and analyzed the data. FM and MaM, created the idea, managed the team, and finalized the draft.

### Conflict of Interest Statement

The authors declare that the research was conducted in the absence of any commercial or financial relationships that could be construed as a potential conflict of interest.
